# Pediatric out-of-hospital cardiac arrest in Denmark

**DOI:** 10.1186/s13049-022-01045-x

**Published:** 2022-11-17

**Authors:** Mathias Geldermann Holgersen, Theo W. Jensen, Niklas Breindahl, Julie L. B. Kjerulff, Sara H. Breindahl, Stig Nikolaj Fasmer Blomberg, Signe Amalie Wolthers, Lars Bredevang Andersen, Christian Torp-Pedersen, Søren Mikkelsen, Freddy Lippert, Helle Collatz Christensen, Danish Cardiac Arrest Registry Group

**Affiliations:** 1 Copenhagen Emergency Medical Services, Telegrafvej 5, 2750 Ballerup, The Capital Region of Denmark Denmark; 2 Department of Clinical Medicine, University of Copenhagen Denmark, Copenhagen, Denmark; 3 Department of Paediatrics and Adolescent Medicine, Paediatric Pulmonary Service, Copenhagen, Denmark; 4 Prehospital Center Region Zealand, Næstved, Denmark; 5 Department of Research and Development, Emergency Medical Services, Aarhus, Central Denmark Region Denmark; 6 Department of Neonatal and Pediatric Intensive Care, Copenhagen University Hospital, Rigshospitalet, Copenhagen, Denmark; 7 Departments of Cardiology, Nordsjaellands Hospital, Hillerød, Denmark; 8 Department of Public Health, University of Copenhagen, Copenhagen, Denmark; 9 Prehospital Research Unit, Department of Regional Health Research, University of Southern Denmark, Odense, Denmark; 10 The National Clinical Registries, National Registries, Copenhagen, Denmark; 11 CAMES, Herlev Hospital, Herlev, Denmark

## Abstract

**Background:**

Pediatric out-of-hospital cardiac arrest (POHCA) has received limited attention. All causes of POHCA and outcomes were examined during a 4-year period in a Danish nationwide register and prehospital medical records. The aim was to describe the incidence, reversible causes, and survival rates for POHCA in Denmark.

**Methods:**

This is a registry-based follow-up cohort study. All POHCA for a 4-year period (2016–2019) in Denmark were included. All prehospital medical records for the included subjects were reviewed manually by five independent raters establishing whether a presumed reversible cause could be assigned.

**Results:**

We identified 173 cases within the study period. The median incidence of POHCA in the population below 17 years of age was 4.2 per 100,000 persons at risk. We found a presumed reversible cause in 48.6% of cases, with hypoxia being the predominant cause of POHCA (42.2%). The thirty-day survival was 40%. Variations were seen across age groups, with the lowest survival rate in cases below 1 year of age. Defibrillators were used more frequently among survivors, with 16% of survivors defibrillated bystanders as opposed to 1.9% in non-survivors and 24% by EMS personnel as opposed to 7.8% in non-survivors. The differences in initial rhythm being shockable was 34% for survivors and 16% for non-survivors.

**Conclusion:**

We found pediatric out-of-hospital cardiac arrests was a rare event, with higher incidence and mortality in infants compared to other age groups of children. Use of defibrillators was disproportionally higher among survivors. Hypoxia was the most common presumed cause among all age groups.

## Background

Pediatric out-of-hospital cardiac arrest (POHCA) is a rare event in which management and emergency responses vary among regions [1, 2]. Current practices depend mostly on reviews based on data from past decades. Different definitions of POHCA exist alongside various methods for reporting and documentation while reported incidence rates are ranging from 3.3 to 6.7 per 100,000 persons at risk [3–10]. In general, higher incidence rates are reported for infants compared to other age groups of children [3, 6, 7, 11–13]. Thirty-day survival rates range from 5 to 11% [3, 6, 7, 14–17] and survival until hospital discharge ranges from 2 to 11% [5, 8, 9, 11, 12, 18–20]. Differences might be explained by different age limits in the definition of age groups. The inconsistencies in the definition of age groups within children underline a general trend in POHCA studies in which heterogeneous or missing definitions, reporting templates, and data validation processes complicate comparability and aggregate analyses [21]. Accidents, especially drowning, make up a larger proportion of younger children, while suicide and drug overdoses are seen almost exclusively among teenagers [22–25]. Reporting for individual age groups is crucial and high-quality data sources, including data validation and strict adherence to standardized reporting templates such as the Utstein style for pediatric advanced life support (PALS), are warranted [26, 27].

Focusing on survival, presumed reversible causes are labeled as key components of the advanced resuscitation algorithms [28–34]. Among reversible causes, hypoxia is considered the most common reversible cause of cardiac arrest [13, 34–36]. Compared to adults, POHCA’s are less likely to be primary cardiac events [26, 35] and the etiology of POHCA is often categorized as originating from either a cardiac or non-cardiac trigger [37]. Children with cardiac arrest do not constitute one single patient group, but rather a complicated composition of a population with significant differences. The Danish Cardiac Arrest Registry (DCAR) provide the basis for examining medical reports for reversible causes and can be coupled with high-quality full-population data registries.

The aim was to describe the incidence, reversible causes, survival rates, and relevant characteristics for validated POHCA during a 4-year period in Denmark.

## Methods

This was a registry-based follow-up cohort study, in which all POHCA registered in the DCAR from January 1st, 2016, through December 31st, 2019, were reviewed [38, 39].

### Setting

The Danish population is approximately 5.8 million inhabitants with < 19% aged 0–16 years. Public healthcare is free. The national Emergency Medical Service (EMS) system is divided into five health care regions, using the same national prehospital electronic patient records [40]. In cases of suspected POHCA, an ambulance and an anesthesiologist-paramedic staffed mobile critical care unit are dispatched [41]. Most areas are accessible with response times below 20 min [39]. In Denmark, resuscitation is started on all cases with the exemption of cases presenting with a “do not resuscitate order” or cases with lesions obviously incompatibly with life (e.g. head separated from body). The termination of resuscitation is strictly on the orders from a physician and has no unambiguous criteria but depends on an assessment of the situation in question. All Danish citizens have a unique civil personal registration number (CPR-Number) that includes information on age and sex, with unambiguous individual-level record linkage of Danish registers [42].

### Data source

The DCAR is a national registry containing data on OHCAs in Denmark. The registry contains approximately 5000 annual cases [38, 39]. When responding to OHCA, EMS personnel fill out an electronic record form. To identify cases both registration from EMS personnel and advanced text-searching algorithm are used. Subsequently a validation team manually reviews all the forms and electronic health reports aiming at increasing the data quality.

### Inclusion of cases

**Fig. 1:**
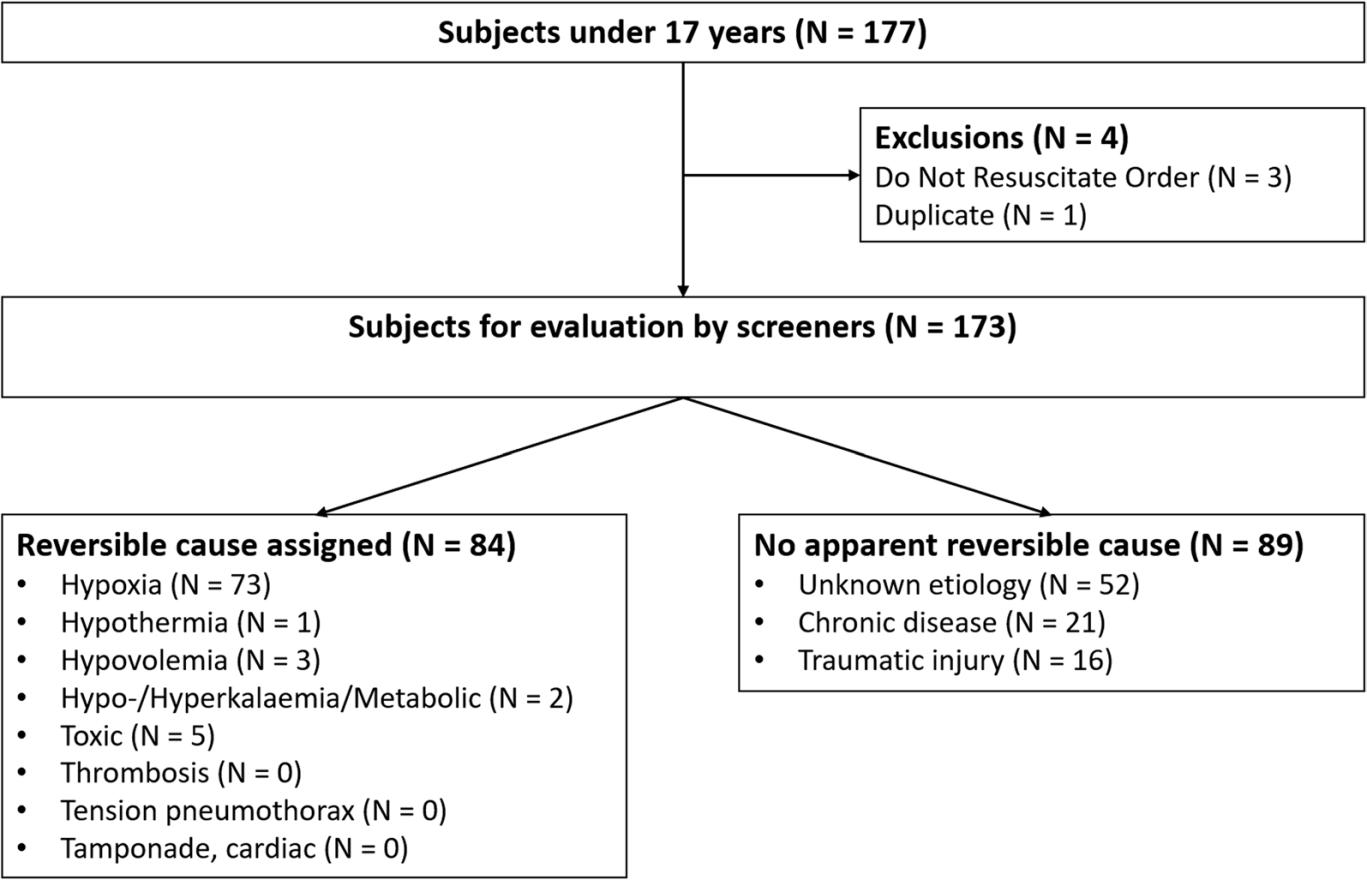
Modified CONSORT diagram. In- and exclusion of subjects including allocation of presumed reversible causes assigned by independent raters reading the prehospital medical charts. Lesions incompatible with life consists of obviously fatal injuries that are not compatible with continued life for which resuscitation efforts are not performed (e.g., the head separated from the body). In 16 cases caused by trauma resuscitation efforts were performed without raters able to identify a presumed reversible cause. Of these 69% (N = 11) were head injuries

Registry data from Danish Cardiac Arrest Registry for out-of-hospital cardiac arrest [38]was extracted from January 1st, 2016, through December 31st, 2019 on subjects below 17 years or with missing age. In some rare instances, the CPR-Number is missing, and the manual labeling served as a secondary control to include all cases under 17 years. Further information on the thirty-day survival rate was retrieved from the Danish Civil Registration System [42]. Cases with “do not attempt resuscitation” (DNAR) orders where brief resuscitation attempts had been initiated by the EMS personnel were excluded (Fig. 1).

### Identification of presumed causes

Five independent raters were tasked to review the prehospital medical records of all cases aimed at labeling a suspected cause of the arrest, and to establish whether there was a presumed reversible cause of POHCA in each case if possible. The raters limited plausible reversible causes to the four H’s and four T’s and indicated only the most prominent, i.e., a single factor, for each case. The raters were physicians or medical students on the last year of study and part of the DCAR validation group. Disagreement was resolved by a third-party senior member from the committee of the DCAR by discussion. Cases presenting without information indicating a reversible were denoted as “No apparent reversible cause”.

### Statistical analysis and presentation of data

Descriptive statistics are presented as numerical counts and percentages for categorical data and as medians including interquartile ranges for continuous data. Statistics are presented for the total cohort as well as stratified age groups; infants (subjects below 1 year of age), pre-school children (1–5 years of age), school children (6–12 years of age), and teenagers (13–16 years of age). Interrater reliability was assessed as Fleiss’ kappa. Comparative analyses addressing age group differences were performed using Pearson’s Chi-square/Fisher’s exact test for proportions while Kruskal Wallis H-test/Mann–Whitney U-test was used for comparing means. *P*-values below 0.05 were considered statistically significant. All analyses were performed using R version 3.6.3.

### Data storage and ethical considerations

The study followed Danish General Data Protection Regulation and was registered and approved by the Danish Data Protection Agency (reference: 2007-58-0015, GEH-2014–019, I-suite 02,737). As this is a registry-based study, ethical approval was not required per present Danish legislation. The protocol was registered at ClinicalTrials.gov (identifier: NCT04275856).

## Results

In the period from January 1st, 2016, through December 31st, 2019, a total of 173 POHCAs were identified in DCAR, which corresponds to 0.8% of all out-of-hospital cardiac arrests recorded in the inclusion period. (Fig. 1). The median incidence of pediatric OHCA was 4.2 (IQR: 3.9–4.2) per 100,000 persons at risk (data not shown). The thirty-day survival rate from POHCA was 40%. Response time ranged from one to 20 min (data not shown), with a median of 7 (IQR: 5–9) min (Table 1). Also seen in Table 1, in 95% (N = 164) of all cases a prehospital physician was present and part of the treatment. In the remaining 5% (N = 9) EMS personnel including paramedics treated the POHCA. The POHCA was witnessed by a bystander in 49% (N = 84) of cases and by EMS personnel in 5% (N = 8) of cases. Bystander defibrillation ranged from 0 to 18% depending on the age group (0% in infants) (Table 2).

**Table 1:** Characteristics of thirty-day survivors and non-survivors of out-of-hospital cardiac arrest in Denmark from 2016 to 2019

Characteristic	Thirty-day survival			*P*-value
	Died (N = 103)	Survived (N = 70)	Overall (N = 173)	
Age, years	3.6 (0.2, 13.3)	6.4 (1.8, 13.4)	5.1 (0.4, 13.4)	0.173
Sex, male	66 (64.1%)	42 (60.0%)	108 (62.4%)	0.587
Response time (minutes)	7.0 (5.0, 9.0)	6.5 (4.0, 9.0)	7.0 (5.0, 9.0)	0.281
Location				< 0.001
Private home	81 (78.6%)	31 (44.3%)	112 (64.7%)	
Nature area	6 (5.8%)	5 (7.1%)	11 (6.4%)	
Traffic area	8 (7.8%)	13 (18.6%)	21 (12.1%)	
Other	8 (7.8%)	21 (30.0%)	29 (16.8%)	
Witnessed by EMS	7 (6.8%)	1 (1.4%)	8 (4.6%)	0.145
CPR by EMS	101 (98.1%)	41 (58.6%)	142 (82.1%)	< 0.001
Defibrillation by EMS	8 (7.8%)	17 (24.3%)	25 (14.5%)	0.002
Witnessed by bystander	35 (34.0%)	49 (70.0%)	84 (48.6%)	< 0.001
CPR by bystander	82 (79.6%)	66 (94.3%)	148 (85.5%)	0.007
Defibrillation by bystander	2 (1.9%)	11 (15.7%)	13 (7.5%)	< 0.001
Medical doctor present	99 (96.1%)	65 (92.9%)	164 (94.8%)	0.488
Initial rhythm shockable	16 (15.5%)	24 (34.3%)	40 (23.1%)	0.004
ROSC before arrival at hospital	26 (25.2%)	70 (100.0%)	96 (55.5%)	< 0.001
State at arrival to hospital				< 0.001
Dead	53 (51.5%)	0 (0.0%)	53 (30.6%)	
Continuous CPR	28 (27.2%)	3 (4.3%)	31 (17.9%)	
ROSC, GCS < 8	20 (19.4%)	32 (45.7%)	52 (30.1%)	
ROSC, GCS > = 8	2 (1.9%)	35 (50.0%)	37 (21.4%)	
Reversible cause	41 (39.8%)	43 (61.4%)	84 (48.6%)	0.005

Values are reported as N (percentage) for count data and as Median (interquartile range) for numeric values

**Table 2:** Characteristics according to age-group in persons with out-of-hospital cardiac arrest in Denmark from 2016 to 2019

Characteristic	> 1 year (Infants) (N = 52)	1–5 years (Preschool) (N = 41)	6–12 years (School) (N = 33)	13–16 years (Teenagers) (N = 47)	*P*-value
Age, years	0.1 (0.0, 0.3)	2.6 (1.8, 4.2)	9.8 (8.6, 11.3)	15.0 (14.4, 16.1)	< 0.001
Sex, male	22 (42.3%)	32 (78.0%)	20 (60.6%)	34 (72.3%)	0.002
Response time (minutes)	7.0 (4.0, 9.0)	7.5 (4.8, 11.0)	7.0 (5.0, 9.0)	7.0 (5.0, 10.0)	0.719
Location					< 0.001
Private home	51 (98.1%)	27 (65.9%)	14 (42.4%)	20 (42.6%)	
Nature area	0 (0.0%)	2 (4.9%)	3 (9.1%)	6 (12.8%)	
Traffic area	1 (1.9%)	1 (2.4%)	5 (15.2%)	14 (29.8%)	
Other	0 (0.0%)	11 (26.8%)	11 (33.3%)	7 (14.9%)	
Witnessed by EMS	1 (1.9%)	3 (7.3%)	1 (3.0%)	3 (6.4%)	0.583
CPR by EMS	45 (86.5%)	32 (78.0%)	25 (75.8%)	40 (85.1%)	0.504
Defibrillation by EMS	3 (5.8%)	4 (9.8%)	6 (18.2%)	12 (25.5%)	0.030
Witnessed by bystander	24 (46.2%)	18 (43.9%)	21 (63.6%)	21 (44.7%)	0.288
CPR by bystander	44 (84.6%)	35 (85.4%)	30 (90.9%)	39 (83.0%)	0.828
Defibrillation by bystander	0 (0%)	1 (2.4%)	6 (18.2%)	6 (12.8%)	0.002
Medical doctor present	49 (94.2%)	38 (92.7%)	33 (100%)	44 (93.6%)	0.519
Initial rhythm shockable	11 (21.2%)	8 (19.5%)	6 (18.2%)	15 (31.9%)	0.406
ROSC before arrival at hospital	20 (38.5%)	29 (70.7%)	18 (54.5%)	29 (61.7%)	0.013
State at arrival to hospital					0.145
Dead	20 (38.5%)	7 (17.1%)	10 (30.3%)	16 (34.0%)	
Continuous CPR	13 (25.0%)	7 (17.1%)	6 (18.2%)	5 (10.6%)	
ROSC, GCS < 8	11 (21.2%)	13 (31.7%)	10 (30.3%)	18 (38.3%)	
ROSC, GCS > = 8	8 (15.4%)	14 (34.1%)	7 (21.2%)	8 (17.0%)	
Thirty-day survival	14 (26.9%)	20 (48.8%)	17 (51.5%)	19 (40.4%)	0.081

Values are reported as N (percentage) for count data and as Median (interquartile range) for numeric values

### Survivors and non-survivors

Table 1 shows different characteristics of the POHCA divided into thirty-day survivors and non-survivors. Median age did not differ between survivors and non-survivors. Similarly, no significant difference in response time was found between survivors and non-survivors. A significant difference in the initial rhythm between survivors and non-survivors was observed. Survivors had an initial shockable rhythm in 34% (N = 24) compared to 16% (N = 16) in non-survivors. The defibrillation rate by bystanders with an AED and by EMS personnel was significantly higher among survivors. Survivors were defibrillated by an AED in 16% (N = 11) of cases as opposed to only 2% (N = 2) for non-survivors. EMS personnel applied a DC shock with a defibrillator in 24% (N = 17) of cases for survivors as opposed to 8% (N = 8) for non-survivors. A significantly higher proportion of POHCA survivors compared to non-survivors were witnessed by bystanders (70% vs 34%) and significantly more survivors than non-survivors received cardiopulmonary resuscitation (CPR) by bystanders (94% vs 80%,). For CPR performed by EMS personnel, there was a significantly higher proportion among non-survivor than survivors (59% in survivors vs 98%in non-survivors). For the non-surviving subjects who received CPR by EMS personnel, 19.8% (N = 20) received no CPR by bystanders. All survivors achieved ROSC before hospital arrival and only 4% (N = 3) were admitted with ongoing CPR.

### Age groups

**Fig. 2:**
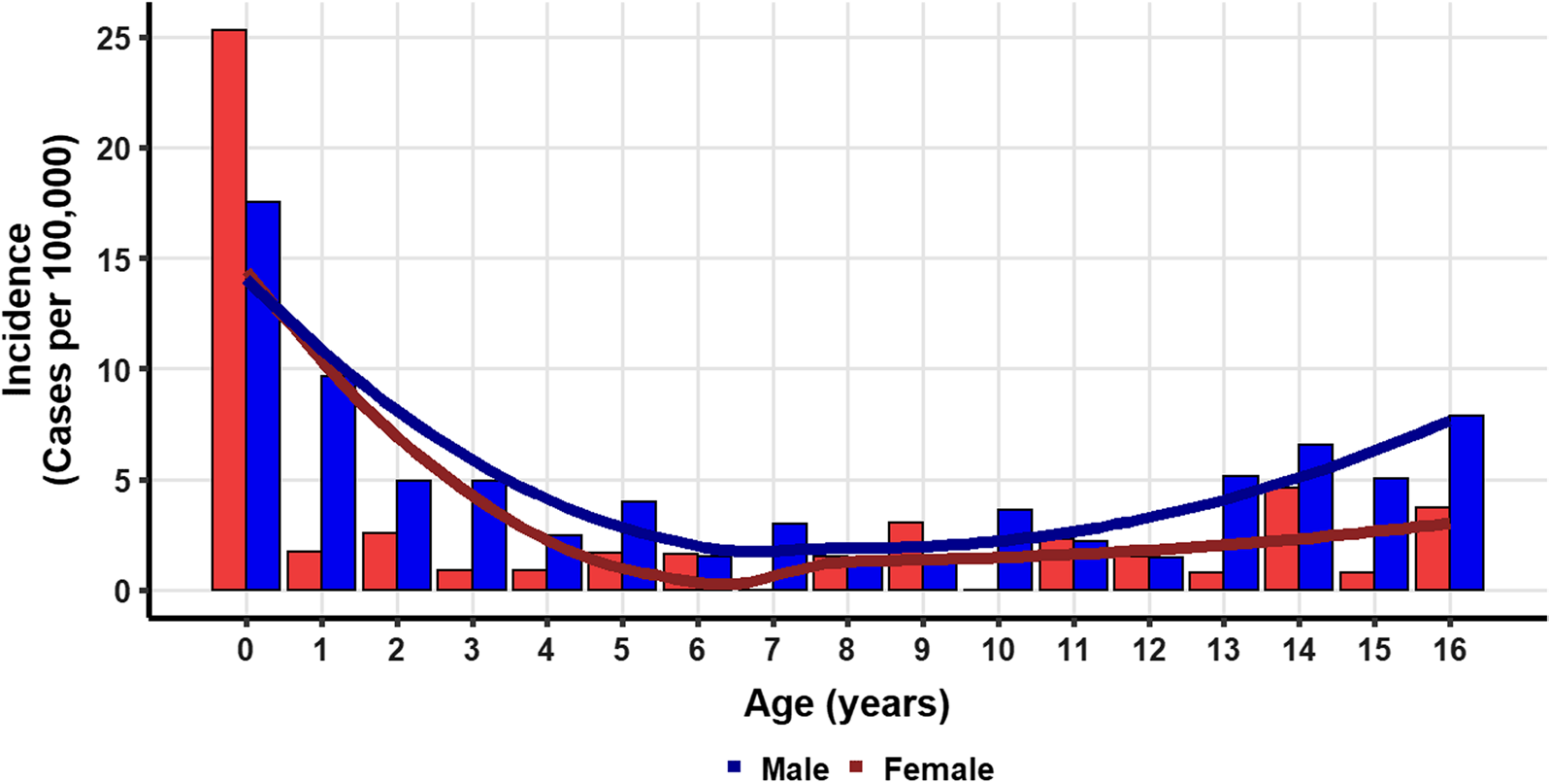
Distribution of age in pediatric out-of-hospital cardiac arrest in Denmark from 2016 to 2019. Incidence according to age for males (blue) and females (red). Solid lines depict smoothed moving averages (dark blue = males, dark red = females)

Table 2 presents cases stratified on age groups. Thirty-day survival did not differ significantly between age groups (*P* = 0.082). However, there was a trend towards lower thirty-day survival among infants (27%, N = 14), compared to the highest seen in school-age children (52%, N = 17). Overall, ROSC was achieved in 55% (N = 96) of cases. Infants had the lowest rate of ROSC (38%, N = 20), whereas preschool children had the highest rate (71%, N = 29). There were significantly more male than female children suffering POHCA in the three oldest age groups (*P* < 0.001, Fig. 2). There was no difference in the initiation of bystander CPR (*P* = 0.8) between age groups. The proportion of cases receiving continuous CPR upon arrival at a hospital ranged from 11 to 25% and did not differ significantly between groups. The group of infants received defibrillation by EMS personnel in 5.8% of cases. The initial cardiac rhythm was shockable in 23% (N = 40) of cases, ranging from 18 to 32%.

### Presumed reversible causes

Interrater reliability was calculated as Fleiss' Kappa scores and ranged from 0.44 (moderate agreement) to 1.0 (perfect agreement), hence showing acceptable levels on all included items. In Table 3, the Fleiss’ Kappa for the five raters is presented. Raters were able to assign a presumed reversible cause in 48.6% (N = 84) of cases. Hypoxia was reported as the presumed reversible cause in 42.2% of cases (N = 73) with a substantial interrater agreement (Fleiss’ Kappa: 0.636). A senior researcher (HCC) decided on the final presumed reversible causes in three cases due to disagreement between raters.

**Table 3:** Allocation of presumed reversible causes of out-of-hospital cardiac arrest in Denmark from 2016 to 2019

Reversible cause	> 1 year (Infants) (N = 52)	1–5 years (Preschool) (N = 41)	6–12 years (School) (N = 33)	13–16 years (Teenagers) (N = 47)	Kappa
Hypoxia	21 (40.4%)	23 (56.1%)	11 (33.3%)	18 (38.3%)	0.64
Hypothermia	0 (0.0%)	1 (2.4%)	0 (0.0%)	0 (0.0%)	1.00
Hypovolemia	0 (0.0%)	0 (0.0%)	1 (3.0%)	2 (4.2%)	0.42
Hypo/Hyperkalemia/Metabolic	0 (0.0%)	0 (0.0%)	0 (0.0%)	2 (4.3%)	0.61
Toxic	0 (0.0%)	1 (2.4%)	0 (0.0%)	4 (8.5%)	0.75
Unknown	31 (59.6%)	16 (39%)	21 (63.6%)	21 (44.7%)	0.59

### Etiologies

Accidents (drowning and trauma) were the etiology in 24% of cases (N = 41). Twenty-four cases (14%) were attributed to drowning incidents while traumatic injuries were linked with 17 (10%) of cases. Suicide was the cause in 7% (N = 12) of all cases, but accounted for 23% of the cases among teenagers, with a median yearly incidence of 1.1 (IQR: 0.9–1.2) per 100.000 person (children) at risk. Only one case of suicide was found among younger age groups. Drug overdoses were only observed as the cause among teenagers and accounted for 4 cases (9%) of cases in the age group.

## Discussion

This is the first study focusing on POHCA in Denmark after the introduction of validation in the DCAR. We found the prevalence of POHCA to be most common in infants and hypoxia to be the leading presumed reversible cause in all age groups. Thirty-day survival rates are 41%. Survival was associated with a higher level of bystander CPR and defibrillation rates.

A previous study by Rajan et al. also based on the DCAR found overall incidence rates to be 3.3 per 100,000, which is comparable to our findings, despite minor differences in age-group definitions [3]. The incidence was found to be highest among infants, though we found a considerably higher incidence (21 vs 12 per 100,000). We found a higher proportion of cases with an initial rhythm that was shockable, (23% vs 12%) and Rajan et al. [3] found substantially lower rates of ROSC upon arrival at the hospital (11% vs. 55%) as well as thirty-day survival rates (8% vs. 41%). The higher rate of ROSC may be related to an unprecedented rise in bystander CPR rates, increasing from an overall 49% to 86% as well as an increase in the use of AEDs by bystanders from 0.3 to 7.5%. This study was conducted in a different period with a range of differences that require further investigations before direct comparisons can be made. We speculate that some of these differences might partly be explained by the introduction of manual validation of the DCAR, including the removal of incorrect entries into the registry as well as an extended inclusion based on automatic entry into the registry following the introduction of electronic health records [38, 39].

In accordance with other studies, we found presumed hypoxia accounted for most POHCA cases with non-traumatic origin [13, 29, 36, 37]. In cases where EMS personnel identify a presumed reversible cause of POHCA, the outcome was improved in terms of achieving ROSC and thirty-day survival. This is most likely owed to confounding by indication, where an indication of a possible treatment effect makes EMS personnel work harder at saving the patient rather than presenting an actual predictor for a good outcome. Such anticipated results strengthen the arguments for continued training and education of relevant personnel in the early recognition of reversible causes. The prevalence of drug overdoses in our data is consistent with previous reports [24]. It was only possible to identify a reversible cause in 40.4% of the cases, whereas the presumed reversible cause remained unknown in 59.6% of the cases. We speculate that limited information is either due to limited information on site, poor documentation or because prehospital physicians might report in in-hospital reporting systems. The limited information in the prehospital medical record, presumably reflect that EMS personnel was not able to establish a reversible cause and hence target treatment. A limited ability to target treatment coincide well with the lower survival rate in the group where a presumable cause was not identified (ROSC in 42%, N = 37 and thirty-day survival in 30%, N = 27).

*S*uicide was found to be a common cause among teenagers, with similar reported incidence rates as international reports [22, 23]. Suicide is a preventable cause of POHCA, and this study underlines the need for continuation and further improvement of campaigns to prevent suicides among teenagers and young adults.

The prehospital medical reports should serve as a supplement in surveillance and early intervention for POHCA. This is particularly of current relevance, as novel analytical methods allow computerized text-analysis of large amounts of data possibly providing new insights.

### Strengths and limitations

The major strength of this study is the manual validation of the national cardiac arrest registry that has been carried out.

It was only possible to assign a presumed reversible cause to 49% of cases. The fact that the remaining cases were not assigned a presumed reversible cause does not necessarily mean that no such cause was present but may reflect that the raters were unable to allocate a cause from the information present in the prehospital medical record. As this is an observational study, the design does not enable the generation of causation and evidence. The conclusions concerning potentially reversible causes are severely limited by the low number of cases. However, we believe that this study contributes with an overview of POHCA.

## Conclusion

POHCA is a rare event with a high occurrence of ROSC and thirty-day survival rate of 40%. Higher rates of initial shockable rhythms among 30-day survivors as well as higher rates of defibrillation by both bystanders and EMS personnel was found. Higher rates of bystander CPR were observed among survivors but were not significant. Incidence and mortality were considerably higher among infants, though there was a lack of bystander defibrillation in this age group. Compared to a previous report from Denmark, survival and bystander CPR among pediatric patients has improved substantially.

## Data Availability

Data are available on reasonable request. Please email the corresponding author to request the relevant data. Please provide the authors of the article with a detailed protocol for the proposed study and supply information about the funding and resources to conduct the study. If appropriate, invite the original author(s) to participate in the reanalysis.
